# The faster internal clock in ADHD is related to lower processing speed: WISC-IV profile analyses and time estimation tasks facilitate the distinction between real ADHD and pseudo-ADHD

**DOI:** 10.1007/s00787-017-0971-5

**Published:** 2017-03-10

**Authors:** Marco Walg, Gerhard Hapfelmeier, Daniel El-Wahsch, Helmut Prior

**Affiliations:** 1 SANA-Klinikum, Zentrum für seelische Gesundheit des Kindes- und Jugendalters, Remscheid, Germany; 2 Heinrich-Heine-University, Düsseldorf, Germany; 3 Faculty of Society and Economics, Rhine-Waal University of Applied Sciences, Kleve, Germany; 4 Institute of Psychology, Goethe University, Frankfurt am Main, Germany

**Keywords:** ADHD, Time processing, Retrospective time estimation, WISC-IV profile, Processing speed

## Abstract

Alterations in temporal processing may represent a primary cause of key symptoms in ADHD. This study is aimed at investigating the nature of time-processing alterations in ADHD and assessing the possible utility of testing time estimation for clinical diagnostics. Retrospective verbal time estimation in the range of several minutes was examined in 50 boys with ADHD and 53 boys with other mental disorders. All participants (age 7–16) attended an outpatient clinic for ADHD diagnostics. The diagnostic assessment included the WISC-IV. Subjects with ADHD made longer and less accurate duration estimates than the clinical control group. The ADHD group showed a specific WISC-IV profile with processing speed deficits. In the ADHD group there was a correlation between processing speed and quality of time estimation that was not observed in the comparison group: higher processing speed indices were related to more accurate duration estimates. The findings provide support for the presence of a faster internal clock in subjects with ADHD and lend further support to the existence of a specific WISC-IV profile in subjects with ADHD. The results show that analyzing WISC-IV profiles and time estimation tasks are useful differential diagnosis tools, particularly when it comes to distinguishing between “real ADHD” and pseudo-ADHD.

## Introduction

According to the triple pathway model [46], deficits in temporal processing may represent a primary cause of the key symptoms of ADHD in addition to inhibitory control and delay-related processes. Studies of the association between impulsivity and time estimation could not consistently confirm the hypothesis that deficits in time estimation rely on impulsivity [2, 20, 25, 26]. In clinical studies it is not clear, if time perception may be correlated with impulsivity trait or if it may be correlated with symptoms of the mental disorder [49]. A recent study based on duration discrimination and controlled for a possible impulsivity bias has indicated the presence of a faster internal clock in children with ADHD [51]. Hence, a “pure” time perception deficit in ADHD has been assumed [44] and its use for diagnostics has been discussed [21]. In addition to the duration discrimination method, studies on temporal processing in ADHD have commonly employed reproduction and verbal time estimation tasks [7, 48]. Contrary to studies based on duration discrimination and reproduction tasks, studies on verbal time estimation have yet to yield consistent findings [11].

Verbal time estimation tasks may be conducted with prospective or retrospective estimation. In a prospective estimation task, subjects know in advance that a duration judgment will be required. In a retrospective estimation task, subjects are asked for a judgment without prior warning subsequent to duration presentation [22]. Task demands may differ between these variants. Whereas cognitive resources are primarily allocated to temporal information in the prospective form, more cognitive resources are devoted to non-temporal information in retrospective estimation [59]. Retrospective estimation is also referred to as “remembered duration”, whereas the term “experienced duration” refers to prospective estimation [6].

Studies with prospective verbal time estimation [3–5, 23, 30] have yet to yield significant group differences. McGee, Brodeur, Symons, Andrade and Fahie [29] presented a retrospective estimation task which found that subjects with ADHD made significantly longer and more variable estimates compared to subjects without ADHD. Prevatt et al. [36] researched the time estimation abilities of college students with ADHD in both prospective and retrospective verbal estimation. Compared to students without ADHD, subjects with ADHD overestimated time durations in prospective as well as retrospective testing. In general, earlier studies have provided evidence that suggests that retrospective verbal time estimation is impaired in subjects with ADHD, while indicating there may be no or only slight prospective time estimation impairment.

With regard to clinical samples and the consequences of ADHD in daily life, two neglected but possibly important factors were addressed in the present study. Firstly, most earlier studies compared subjects with ADHD to control subjects without any remarkable psychiatric symptoms. However, the challenge in clinical diagnostics is to distinguish ADHD sensu stricto from other syndromes with partially similar symptoms. If tests of time perception were indeed instrumental in diagnostics [21], it would be important to know whether alterations in temporal processing are specific to ADHD or rather a trans-nosographic phenomenon. For example, Smith et al. [44] found differing results for children with and without ADHD in a reproduction task when IQ and short-term memory were taken into account. This is in line with results from Bauermeister et al. [5] who concluded that impaired time estimation performance in subjects with ADHD may be related to known deficits in inhibition and working memory rather than to a temporal perception deficit. To clarify this issue, participants of the present study were tested with the Wechsler Intelligence Scale for Children (WISC-IV) in addition to retrospective time estimation task to compare time estimation skills with performance on other tasks. Furthermore, the control subjects did not have an ADHD diagnosis, but displayed symptoms such as hyperactivity or impulsivity, which may emulate true ADHD.

Secondly, time estimation tasks were designed to be more relevant to situations in daily life (e.g., at school). These tasks lasted several minutes, whereas most earlier studies had used durations of less than 1 min. This study is the first to use retrospective verbal time estimation tasks in the range of several minutes and the first to employ a combination of time estimation and intelligence diagnostics for profiling children with ADHD and clinical controls that require careful differential diagnostics.

## Methods

### Participants

A total of 113 boys between 7 and 16 years of age participated in this study. All subjects attended the outpatient clinic of a psychiatric hospital for children and adolescents for diagnostic assessment. They all were in suspicion of ADHD because parents and/or teachers reported symptoms of ADHD. Male gender and a diagnosed psychiatric disorder (after diagnostic assessment) were both key inclusion criteria for this study. Total IQs less than 85, any kind of medication and autism spectrum disorder, psychotic disorder, acute and posttraumatic stress disorder, dissociative disorder, eating disorder, or substance-related disorder diagnoses were all key exclusion criteria. Eight participants with a total IQ below 85 were excluded from study after diagnostic assessment. One additional participant was excluded because no psychiatric disorder was diagnosed and another participant was excluded as an outlier as his estimates deviated more than 2 standard deviations from the rest of the sample. The remaining 103 subjects were assigned to the ADHD group or the clinical control group.

Before the beginning of the study, children and their parents had been informed in writing about the study. Informed consent was obtained from all participants.

### Diagnostic assessment

The diagnostic assessment comprised at least four sessions including a semi-structured diagnostic and clinical interview with the child and at least one parent. The interviews were conducted by experienced clinical psychologists or psychiatrists. The psychiatrists conducted physical and neurological examinations for all subjects. The parents completed several rating scales of the *DISYPS*-*II* that measured symptoms of ADHD, depression disorder, and anxiety disorder. The ADHD rating scales were also completed by teachers if the parents agreed. All subjects were tested on the *WISC*-*IV.* School reports were also reviewed. Subjects completed a range of other questionnaires in addition to this standardized assessment (e.g., for symptoms of posttraumatic stress disorder or conduct disorder), specific assessment (e.g., for specific learning disorders), or underwent additional examinations such as EEGs or complete blood count, if such procedures were deemed necessary.

The results from all diagnostic assessments were evaluated by a multidisciplinary team. Members of the team included experienced psychiatrists, psychologists, psychotherapists, and qualified social workers. Most of the evaluators were blinded to the aim of the study and the results of the verbal estimation task.

### WISC-IV

The German version of the Wechsler Intelligence Scale for Children—Fourth Edition (WISC-IV) [35] was used by trained and experienced clinical psychologists. In addition to a total IQ score, the WISC-IV provides index scores for verbal comprehension, perceptual reasoning, working memory and processing speed. The internal consistency reliability coefficient for the full scale is *r* = .97. The reliability coefficients of the four index scores range from *r* = .87 to *r* = .94.

### Rating scales (DISYPS-II)

The diagnostics system for mental disorders in childhood and adolescence (DISYPS-II) [16] consists of rating scales for ADHD, depressive disorders, anxiety disorders (including obsessive–compulsive disorder), conduct disorders, and autism spectrum disorders in childhood and adolescence. It includes scales for self-assessments for subjects 11 years and older as well as scales for ratings by parents, caregivers and teachers. The reliability coefficients of the respective rating scales range from *α* = 0.70 to *α* = 0.90. The ADHD rating scale includes 20 items describing symptom criteria according to ICD-10 and DSM-IV as well as additional items assessing symptom onset, symptom duration and pervasiveness. The rating scale includes 9 items that assess the severity of inattention according to ICD-10 and DSM-IV. The severity of hyperactivity is assessed by 7 items. Since the conceptualization of items is not consistent between DSM-IV and ICD-10, the diverging items are enclosed (symptom of being “on the go” from DSM-IV and symptom of a persistent pattern of excessive motor activity from ICD-10). There is an extra item for the symptom of subjective feelings of restlessness. The severity of impulsivity is assessed by the 4 items according to ICD-10 that include the symptom of talking excessively. There is empirical evidence for the validity of the three-factor solution comprising inattention, hyperactivity and impulsivity separately [19]. A comparison study of the ADHD rating scale with the Conner’s scale has shown that both instruments are suitable in the assessment of ADHD [18].

### Retrospective verbal time estimation

The time required to complete the subtest *Matrix Reasoning* (from first to last test item) was measured while the WISC-IV test was administered. Upon completing the subtest, the subjects were asked to estimate in minutes and seconds how long it had lasted. No prior indication that a judgment of the subtest duration would be required had been provided.

Time estimation was calculated as the relative (percentage of) deviation of individual estimates from the actual duration. Based on these values, systematic errors as well as accuracy scores were used to compare the estimation performance between groups and to assess correlations with other measures. The systematic error is the signed (+/−) difference between estimated and real task durations, meaning this measure accounts for the direction of differences (over- and underestimates). The accuracy score is the absolute (unsigned) value of the deviation [8], which shows the magnitude of a participant´s error irrespective of the direction.

### Statistical analyses

Descriptive statistics with computations of means (with standard errors) were used for all measures, including WISC-IV indices, total scores and subscale scores of DISPYS-II, and measures of time estimation. Variance analyses were conducted to determine differences between groups. Pearson correlations were carried out to investigate associations between variables. All analyses were conducted using the Statistical Package for the Social Sciences (SPSS for Windows, version 14).

## Results

As shown in Table 1, the ADHD group (mean age = 10.2 years; median age = 10 years; interquartile range = 2 years) included 29 subjects with combined presentation (ADHD-C) and 21 subjects with predominantly inattentive presentation (ADHD-PI). The clinical control group consisted of 53 subjects with depressive disorders, anxiety disorders, adjustment disorders, specific learning disorders, reactive attachment disorders or conduct disorders (mean age = 10.8 years; median age = 11 years; interquartile range = 4 years). In spite of slightly higher average age in the control group, age did not differ significantly between groups (Mann–Whitney U test, *p* = 0.13).

**Table 1:** Mean age (with SD) and frequencies of mental disorders in the ADHD group and in the clinical control group

	ADHD group (*n* = 50)	Control group (*n* = 53)
Age	10,2 (1,9)	10,8 (2,1)
ADHD combined presentation	29	0
ADHD inattentive presentation	21	0
Anxiety disorder	0	12
Depressive disorder	0	5
Adjustment disorder	0	13
Reactive attachment disorder	0	2
Specific learning disorder	0	10
Conduct disorder	0	11

The subtest *Matrix Reasoning* was completed with a mean duration of 5 min and 30 s (SEM = 14 s). There was no group difference in the time required for completion [*F*(1,103) = 0.34, *p* = 0.56]. The results of the verbal time estimation tasks, WISC-IV and DISYPS-II are shown in Table 2.

**Table 2:** Verbal time estimation, WISC-IV, and DISYPS-II results (rated by parents and teachers)

Measure	ADHD	Other	Group difference	Cohen’s *d*
Time estimation				
Accuracy score (%)	59.20 ± 6.74	33.50 ± 4.80	*p* = 0.002	0.62
Systematic error (%)	42.36 ± 8.96	2.99 ± 6.66	*p* < 0.001	0.70
WISC-IV				
Full-scale IQ	101.32 ± 1.49	102.09 ± 1.26	–	−0.08
Verbal comprehension	105.24 ± 1.95	103.49 ± 1.37	–	0.15
Perceptual reasoning	104.68 ± 1.56	101.74 ± 1.34	–	0.28
Processing speed	92.54 ± 1.71	103.98 ± 1.31	*p* < 0.001	−1.06
Working memory	99.72 ± 1.52	98.04 ± 1.56	–	0.15
DISYPS-II				
ADHD total score (parents)	31.94 ± 1.74	22.55 ± 1.71	*p* < 0.001	0.76
Inattention (parents)	17.96 ± 0.78	12.42 ± 0.89	*p* < 0.001	0.93
Hyperactivity (parents)	8.38 ± 0.78	5.54 ± 0.70	*p* = 0.008	0.54
Impulsivity (parents)	5.60 ± 0.52	4.58 ± 0.48	–	0.28
ADHD total score (teacher)	30.00 ± 2.38	21.22 ± 1.94	*p* = 0.008	0.73
Inattention (teacher)	18.29 ± 0.91	11.30 ± 1.13	*p* < 0.001	1.25
Hyperactivity (teacher)	7.12 ± 1.17	5.19 ± 0.71	–	0.37
Impulsivity (teacher)	4.59 ± 0.73	4.78 ± 0.78	–	−0.05
Depression (parents)	13.73 ± 1.58	13.90 ± 1.46	–	−0.02
Anxiety (parents)	13.51 ± 1.36	15.69 ± 1.44	–	−0.22

Means and SEM. There was a strong difference in both time estimation measures between subjects with ADHD and subjects with other mental disorders. Effect sizes are given as Cohen’s *d.* Positive values of *d* indicate higher mean values in the ADHD group

**Fig. 1:**
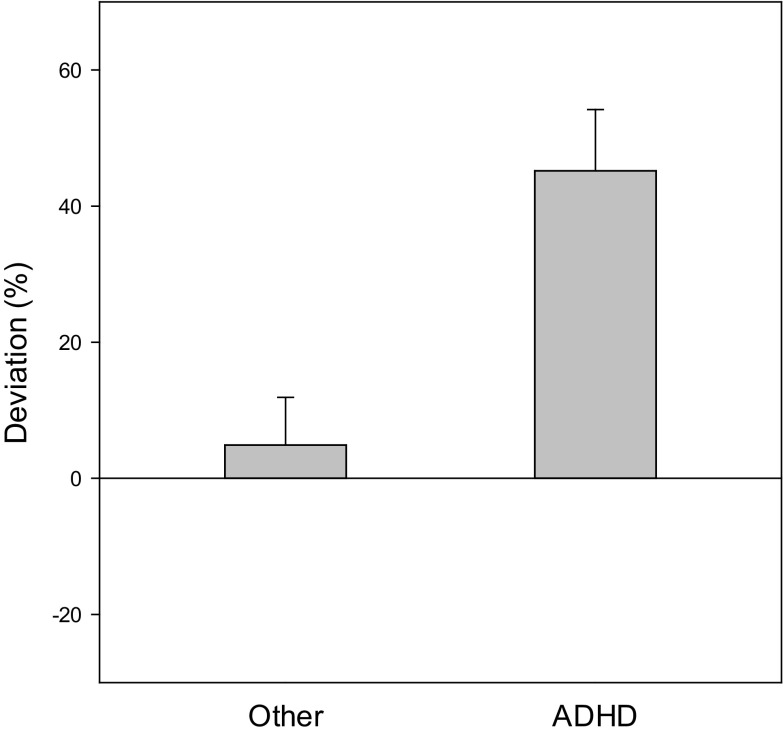
Systematic error. Average estimates were close to actual durations (*p* = 0.47) in patients with other conditions, whereas overestimation was observed in children with ADHD (*p* < 0.001). The two groups differed significantly (*p* < 0.001)

**Fig. 2:**
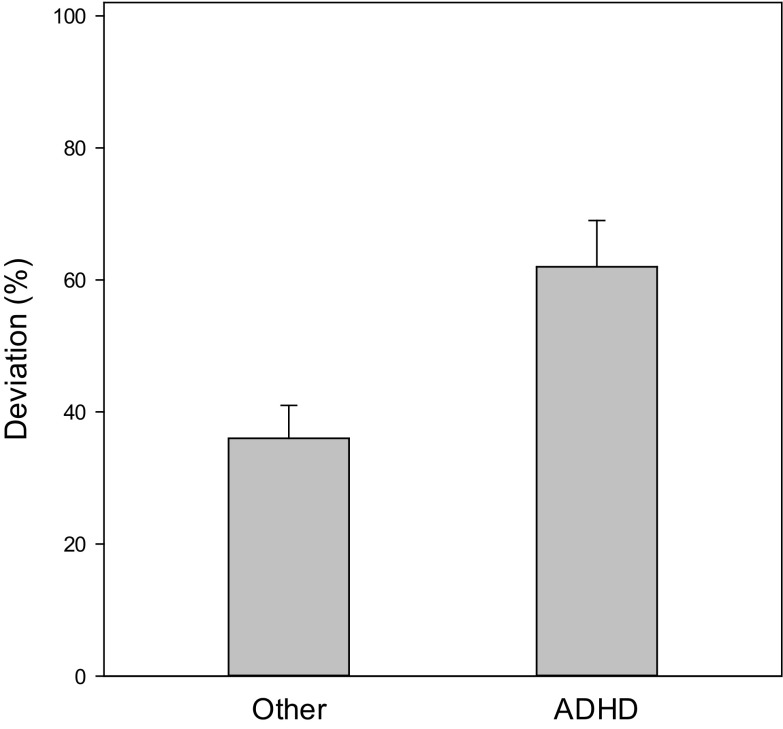
Accuracy score. Overall deviations, irrespective of direction, were greater in patients with ADHD (*p* = 0.003)

Subjects with ADHD gave significantly higher time estimates than subjects with other mental disorders (*p* < 0.001). Figure 1 shows the systematic error in the ADHD group as compared to the clinical control group. A pronounced overestimation was observed in subjects with ADHD, whose estimated durations were approximately 40% longer than actual duration times. The average estimates of subjects in the control group were closer to the actual durations. Consequently, subjects with ADHD have higher accuracy scores as compared to subjects with other mental disorders (see Fig. 2), meaning estimates in the ADHD group were less accurate than estimates in the clinical control group (*p* < 0.003). Separate analysis of subtypes showed that subjects with ADHD-C (*p* = 0.005, *d* = 0.61) and subjects with ADHD-PI (*p* = 0.004; *d* = 0.83) showed significantly longer estimates than controls (means and SEM: ADHD-C 38.40 ± 12.43%; ADHD-PI 47.83 ± 12.94%; controls 2.99 ± 6.66%). Likewise, accuracy scores differed significantly from controls in the combined presentation (*p* < 0.001, *d* = 0.66) as well as in the predominantly inattentive presentation (*p* = 0.004, *d* = 0.59) (means: ADHD-C 58.46 ± 9.23%; ADHD-PI 60.22 ± 10.02%; controls 33.50 ± 4.80%). Between ADHD subtypes, there were no significant differences (systematic error, *p* = 0.61; accuracy score, *p* = 0.90).

The results of the WISC-IV showed a significant difference in processing speed between groups. Subjects with ADHD had lower processing speed indices than subjects with other mental disorders [*F*(1,105) = 28.51, *p* < 0.001]. There are no differences between the two groups in terms of full-scale IQ, verbal comprehension, perceptual reasoning or working memory. Separate analysis of processing speed in subtypes showed that both ADHD-C (*p* < 0.001, *d* = −0.77) and ADHD-PI (*p* < 0.001; *d* = −1.51) showed significantly lower processing speed indices than controls (means and SEM: ADHD-C 95.86 ± 2.14; ADHD-PI 87.95 ± 2.53; controls 103.98 ± 1.31). The difference between ADHD subtypes with lower processing speed in ADHD-PI was also significant (*p* = 0.021; *d* = 0.68).

**Fig. 3:**
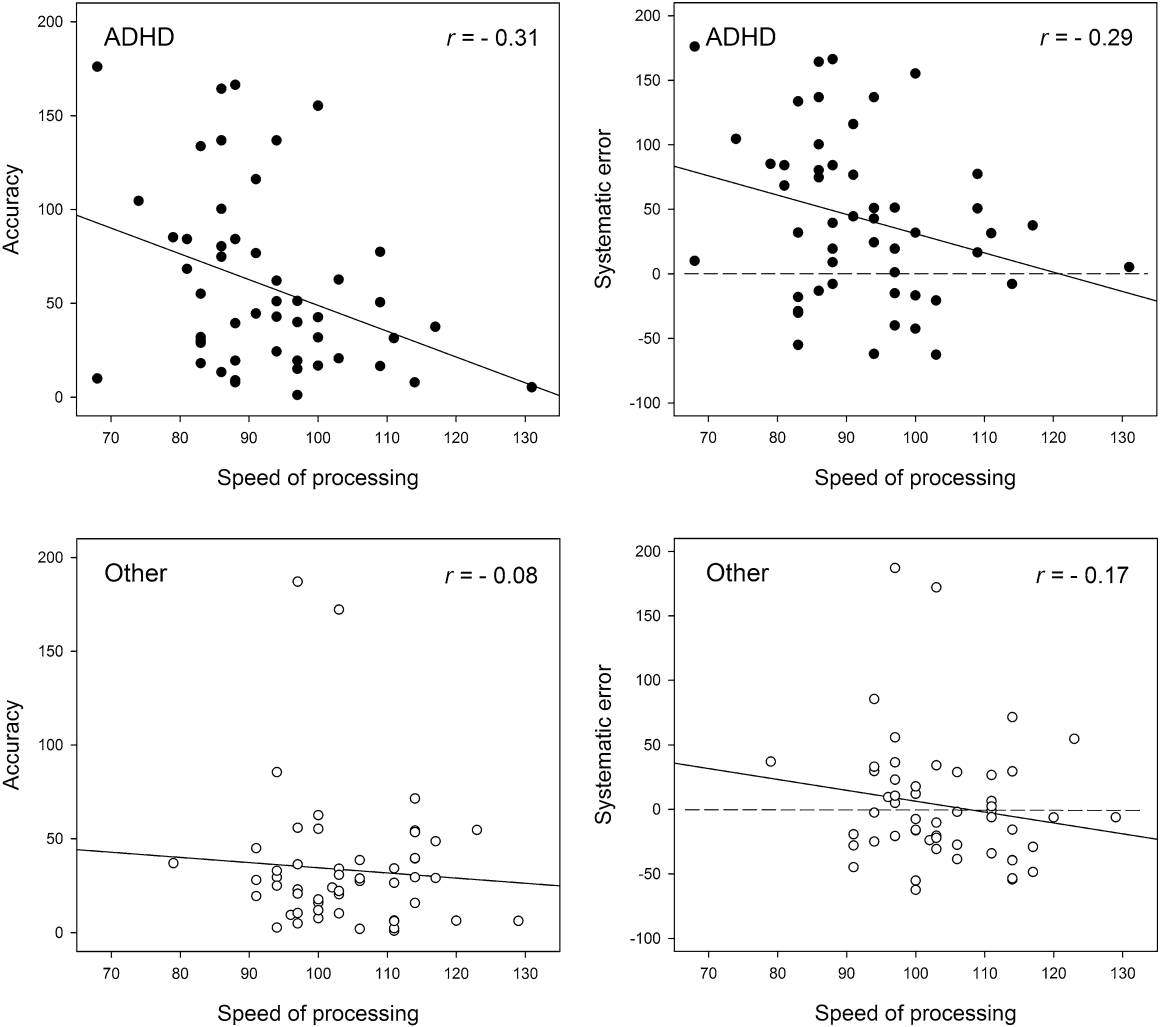
Processing speed, accuracy scores, and systematic estimation errors in children with ADHD and children with other psychiatric disorders. In ADHD, there was a significant correlation between processing speed and overall estimation accuracy as well as between processing speed and systematic error. Subjects with high processing speed indices provided more accurate estimations as indicated by *smaller errors* (*p* < 0.05). *Dashed lines* indicate the level of correct estimates

The relation between time estimation and processing speed in both groups is shown in Fig. 3. In the ADHD group, there are correlations between processing speed and systematic error as well as between processing speed and accuracy score. Subjects with a lower processing speed index made longer and less precise estimates. No such correlation was found for the clinical control group.

Post hoc calculations of achieved statistical power indicated a power of 0.93 for the accuracy score, 0.97 for the systematic error, and >0.99 for processing speed.

Ratings by parents in the ADHD group yielded higher ADHD total scores compared to the clinical control group [*F*(1,105) = 16.51, *p* < 0.001] as well as higher scores on the inattention [*F*(1,105) = 22.96, *p* < 0.001] and hyperactivity subscales [*F*(1,105) = 8.84, *p* = 0.004]. Teachers’ ratings also yielded higher ADHD total scores [*F*(1,63) = 8.56, *p* = 0.005] and higher scores on the inattention subscale [*F*(1,63) = 27.49, *p* = 0.001] for the ADHD group. No significant differences were measured between the two groups on the impulsivity subscale or in the total scores for depression and anxiety.

A possible effect of age on any of the reported effects was assessed by means of ANCOVA. There were no contributions of age to any of the observed group differences, except for the ratings by parents on the inattention subscale. These rating scores increased with higher age of children in the control group (*r* = 0.32; *p* = 0.018), but not in the ADHD group (*r* = 0.08; *p* = 0.60).

## Discussion

Retrospective time estimates of subjects with ADHD were significantly longer and less accurate than estimates made by patients with other psychiatric disorders. The WISC-IV results showed a specific profile for the ADHD group, which had significantly lower processing speed indices compared to the clinical control group. No differences were observed between groups for the other index scores or full-scale IQ. In terms of the ADHD group, there were correlations between processing speed index and systematic error as well as between processing speed index and accuracy score: subjects with higher scores in processing speed provided estimates of relevant time durations that were shorter and more precise.

The findings of the present study have implications for diagnostic assessment in clinical practice: administering the WISC-IV and an additional time estimation task provides useful information for differential diagnostics, in particular in terms of distinguishing true cases of ADHD from pseudo-ADHD. Since there is no single instrument that can provide a definitive ADHD diagnosis, several methods should be used within the diagnostic process. Rating scales have a prominent role in this process and are reliable in terms of differentiating between subjects with ADHD and subjects without psychiatric disorders. However, there are often discrepancies among ratings of child psychopathology provided by parents, children and teachers [15]. Distinguishing subjects with ADHD from subjects with another psychiatric disorder is more difficult. Children suffering from emotional disorders such as depression can show the core symptoms of ADHD, namely attention deficits, hyperactivity and impulsivity [12, 14]. Subjects with internet addiction also show symptoms of ADHD, depression and anxiety [58]. Children with ADHD are at increased risk for developing depressive symptoms [13]. This overlap of symptoms across several psychiatric disorders is also reflected in the results of the rating scales in the present study: there were no significant differences between groups on the impulsivity, depression and anxiety scales. A comparatively low processing speed in the WISC-IV profile and high overestimations of task duration can be considered indicators for ADHD.

The results provide further support for the existence of a specific WISC-IV profile for subjects with ADHD. A performance profile with a weakness in processing speed occurs not only compared to non-clinical controls [27], the current findings show that this specific weakness is also apparent compared to a clinical control group. This may be relevant for diagnostic assessment since distinguishing ADHD from other psychiatric disorders (e.g., affective disorders) is more challenging in clinical practice than distinguishing children with ADHD from those without any disorder. There is evidence to suggest that a significant weakness in processing speed occurs in ADHD subtypes [10] and in ADHD with co-occurring affective disorders or oppositional-defiant disorder [28]. The results of the present study confirm findings of lower processing speed indices in subjects with ADHD-PI in comparison to subjects with ADHD-C [10, 57]. Additional weaknesses in the working memory indices for subjects with ADHD appear to emerge only in cases where comorbid disorders are present [43]. Walg et al. [52] have discussed the benefits of WISC-IV profile analysis as a diagnostic tool.

ADHD is associated with deficits in a variety of executive functions [54]. Alterations in time interval estimations seem to be a core cognitive deficit [45] that is characterized by higher subjective internal clock speeds [24, 40, 47]. The findings of the present study provide support for the presence of a faster internal clock in children with ADHD. This applies to both ADHD subtypes: the combined presentation and the predominantly inattentive presentation. This faster internal clock may lead to overestimations in retrospective verbal time estimation tasks. Previous studies have shown that subjects with ADHD have deficits in retrospective time estimation in the range of seconds [32]. The present study shows that these deficits also emerge in the range of several minutes.

The findings of the present study indicate that alterations in retrospective time estimation are more likely based on pacemaker speed or reference memory. There are different, partially incompatible psychological and neurological conceptualizations of temporal processing [55]. Most psychological models assume there is an internal pacemaker–accumulator clock [9]. During a given time interval, pulses (or “clock ticks”) are produced by a pacemaker and collected by an accumulator. The content of the accumulator represents the experienced time duration and can be transferred from working memory to reference memory for long-term storage and duration judgments. Deficits in time perception may result from alterations in attention processes, pacemaker speeds or in memory. Earlier studies have provided evidence that subjects with ADHD show alterations in prospective timing when attention is directed to the passage of time due to attention deficits [1]. In retrospective timing, attention is not directed to the passage of time, so attention processes should not primarily be responsible for alterations in retrospective time estimation in the ADHD group. Because no differences in working memory index were found between the two groups, there is no evidence to suggest that deficits in working memory resulted in higher and less accurate estimates. These results are in line with findings by Smith et al. [44], who also concluded that it is unlikely that alterations in temporal processing in ADHD can be accounted for by deficits in working memory. Since subjects in the ADHD group performed equally well on the WISC-IV with the exception of processing speed, it is not plausible that the reported group differences should be attributed to motivational deficits. It is therefore more likely that the reported differences in retrospective time estimations are based on alterations in pacemaker speed or reference memory.

Investigating the interdependence of time perception and processing speed and their underlying factors is difficult since both processes are highly complex. Processing speed in the WISC-IV involves, for example, mental speed, reaction time, choice reaction time, visual scanning, learning new material, recall abilities and motor speed [53]. Processing temporal information involves various levels of analysis, from simply perceiving the passage of a short duration to higher cognitive processes such as planning or anticipating. The specific neurophysiological mechanisms and cognitive processes for the inner representation of time have as yet to be completely identified. The correlation between the processing speed index and time estimation accuracy suggests the same processes and regions in the brain are involved. Findings by Droit-Volet and Zélanti [17] indicated that higher processing speed indices are proportional to higher time sensitivities. They note that information processing speed is a better predictor of prospective temporal accuracy than working memory or selected attention. The results of the present study show that this finding also holds true for the retrospective paradigm. Droit-Volet and Zélanti concluded that processing speed and temporal processing are interdependent because processing speed tests go hand in hand with temporal constraints.

Processing temporal information is crucial for performing effectively in everyday life, in particular in terms of predicting, anticipating, and responding efficiently in a variety of situations, for example, when it comes to being on time for an appointment, planning in an appropriate amount of time in which to complete homework assignments or scheduling various other daily tasks. Rammsayer and Brandler [38] assumed that temporal information processing performance constitutes an index of general intelligence, namely *temporal g*. Findings for time-processing alterations in children with ADHD correspond with frequent reports from parents that these children have a poor perception of time and time-related problems in everyday activities [37]. Mioni et al. [31] showed that time perception performance predicts deficits in time-based prospective memory, which is the ability to remember to perform an intended action at a given time in the future. Children with ADHD were less accurate in time-based prospective memory tasks than children without ADHD. Difficulties in retrospective time estimation in particular appear to be related to deficits in academic functioning in the college setting [36] and temporal processing deficits are still present in adults with ADHD [50]. This may account for observed difficulties in professional life. ADHD is also associated with systematic biases in temporal orientation that contribute to functional problems such as difficulties with planning and time management [11]. A faster internal clock in ADHD may result in reinforcing effects that wane more quickly [42], which may result in discounting the value of delayed rewards [56]. Immediate reinforcement as a means of shaping behavior is thus very important in terms of educating and parenting children with ADHD. Therapies of ADHD, such as attention trainings, should consider both the improvement of processing speed as well as training time estimation. There is also evidence to suggest that music training increases processing speed [39]. Improving time estimation competence and increasing the *temporal g* may manifest itself the form of more self-control and a greater appreciation of delayed rewards, better planning and organization skills and thus improved performance in everyday life.

This study has some limitations. The small sample size precludes a serious statistical analysis of subgroups. Future research should investigate retrospective verbal time estimation in homogeneous subject samples with “pure” depressive disorder, anxiety disorder, or conduct disorder and in different presentations of ADHD. The findings of the present study are restricted to male subjects. Barkley et al. [4] did not observe differences in time estimation abilities between male and female subjects with ADHD. Nevertheless, the results for girls should be interpreted with great caution since there is evidence that suggests isolated neuropsychological differences between girls and boys with ADHD [34], including slower processing speeds in affected boys [41] and higher impairments in terms of planning in girls [33].

In conclusion, the findings of the present study confirm deficits in time perception and processing speed in children and adolescents with ADHD. Taking these parameters into account is likely to enhance the validity of diagnostic assessments. Based on the present findings, the analysis of WISC-IV profiles in combination with retrospective time estimation testing is advisable. Likewise, the awareness of alterations in temporal processing in children with ADHD among parents and teachers will help to improve everyday life for those affected.
